# Volunteering, Charitable Donation, and Psychological Well-Being of College Students in China

**DOI:** 10.3389/fpsyg.2021.790528

**Published:** 2022-01-07

**Authors:** Yun Geng, Yafan Chen, Chienchung Huang, Yuanfa Tan, Congcong Zhang, Shaoming Zhu

**Affiliations:** ^1^School of Government, Central University of Finance and Economics, Beijing, China; ^2^School of Social Work, Rutgers, The State University of New Jersey, New Brunswick, NJ, United States; ^3^Research Institute of Social Development, Southwestern University of Finance and Economics, Chengdu, China; ^4^Department of Youth Work Research, China Youth University of Political Studies, Beijing, China; ^5^School of Law, University College Cork, Cork, Ireland

**Keywords:** volunteering, charitable donations, psychological well-being, college students, China

## Abstract

Psychological well-being indicates individuals’ positive psychological functioning and well-being. A growing body of literature, largely based on adults and old people, suggests that volunteering and charitable donations are related to individuals’ psychological well-being. As emerging adulthood is a vital time for lifespan development, the aim of this study is to examine the effects of volunteering and charitable donation on individuals’ psychological well-being on college students. Relying on theories of altruism and the warm-glow theory, this study estimates the relationships among hours of volunteering, amount of charitable donations, and psychological well-being from 1,871 Chinese college students. The linear regression results indicate that students’ hours of volunteering and the amount of charitable donations in the past year were positively associated with their psychological well-being. Volunteering, compared to charitable donations, had a slightly stronger association with psychological well-being. This study provides a rationale for academic institutions to integrate social service activities into the curriculum as a potential tool to promote students’ psychological well-being.

## Introduction

Psychological well-being is a critical indicator of individuals’ positive psychological functioning and general well-being (Ryff and Singer, 1996). Multiple aspects compose psychological well-being, including self-acceptance, positive relations with others, autonomy, environmental mastery, purpose in life, and personal growth (Ryff and Singer, 1996). Self-acceptance emphasizes accepting one’s self and one’s past life as well as holding positive attitudes toward oneself. Positive relations with others denotes individuals’ capacity to have strong empathy, friendship, and affection and perception that they have warm, trusting interpersonal relationships with others. Autonomy stresses self-determination, independence, and the regulation of behaviors. Environmental mastery represents individuals’ competence in manipulating and control environments better for their psychological conditions through physical and mental activities. Purpose in life refers to a strong belief in life’s purpose, a sense of directedness, and intentionality. Lastly, personal growth indicates the continued development of one’s potential through growing and expanding (Ryff and Singer, 1996). Psychological well-being has been shown to have close associations with various positive outcomes, including but not limited to better physical and mental wellness (Keyes, 2005; Kubzansky et al., 2018; Pressman et al., 2019), increased life satisfaction (Boyle et al., 2009), and ideal job performance (Wright and Staw, 1999; Lyubomirsky et al., 2013).

Researchers have developed extensive interests in understanding the factors that contribute to psychological well-being (Wardle and Cooke, 2005; Huppert, 2009; Bewick et al., 2010; Tan et al., 2021). Several factors, such as income, social networks, and optimistic attitudes, are related to individuals’ self-report well-being (Kaplan et al., 2008; Burris et al., 2009; Awaworyi Churchill and Mishra, 2017). Another stream of study focuses on the positive effects of volunteer activities and charitable donations on psychological well-being (Morrow-Howell et al., 2003; Choi and Kim, 2011; Appau and Churchill, 2019). Research consistently suggests that individuals giving their time and money tend to report higher life satisfaction, fewer depressive symptoms, and better overall psychological well-being (Morrow-Howell et al., 2003; McMunn et al., 2009; Choi and Kim, 2011).

The positive association between volunteering and psychological well-being could be comprehended as the reward of volunteering. Many people report a good feeling after helping others (Wuthnow, 1991; Musick and Wilson, 2003). Most people adhere to the value of “making the world a better place” and establish a better feeling about self when acting in the light of their values. Helping others grants our lives a sense of mission, purpose, and meaning. Volunteering can also develop interpersonal trust between individuals who provide help and who receive help, as well as create a sense of security and acceptance for the volunteers. Volunteering behaviors promote social interactions, which provide emotional warmth. In many cases, benevolent acts allow us to take pride in our skills and strengths and promote the sense of self (Musick and Wilson, 2003). Drawn data from three waves of the American’s Changing Lives dataset (House, 1995), Musick and Wilson (2003) found that individuals who had done any volunteer work at the first wave had significantly higher levels of self-esteem at the second wave, compared to those who had not volunteered.

Black and Living (2004) conducted a qualitative study to explore the benefits on individuals’ well-being from volunteering. After analyzing 109 sets of questionnaires returned by volunteers, Black and Living found that participants reported a total of 159 benefits of volunteering, among which 90 were about aspects of psychological health and well-being, and 35 were related to social functioning. Participants perceived volunteering as an opportunity to enhance well-being. Many volunteers experienced positive feelings through helping others, such as rewarding, fun, worthwhile, and satisfying. Some denoted that helping others promoted their spirits as it made them push their own worries aside. Others also denoted obtaining self-confidence and a sense of achievement from others’ acceptance and appreciation. Additionally, volunteering contributed to participants’ social contact and support. Volunteers had the opportunities to establish and maintain close social relationships and a sense of community involvement. These benefits of volunteering ultimately advanced individuals’, particularly volunteers’, psychological health and well-being (Black and Living, 2004).

Charitable donations could be viewed as another type of volunteering that the donors actively involve with charitable organizations (Choi and Kim, 2011). Moreover, studies also revealed that volunteering and charitable donation had some motivational factors in common, such as altruism, a sense of social responsibility, sympathy, and social networks (Small et al., 2007; Apinunmahakul and Devlin, 2008; Dickert et al., 2011; Froyum, 2018). Given the shared motivation for both volunteering and charitable donation, the latter might contribute to individuals’ psychological well-being as the former.

Effects of volunteering and charitable donation on the psychological well-being have been documented by empirical studies (Choi and Kim, 2011; Appau and Churchill, 2019). For instance, Choi and Kim (2011) used the first and second waves of Midlife Development in the United States (MIDUS, Brim et al., 2004) to examine the effects of time volunteering and charitable donation on psychological well-being in later life over a period of 9 years. Accounting for psychological well-being and human, cultural, and social capital resources at the first wave, Choi and Kim (2011) found that a moderate amount of time volunteering (no more than 10 h every month), as well as any amount of charitable donations at the first time point, had positive effects on subjects’ psychological well-being at the later time point. Furthermore, charitable donations had stronger effects than time volunteering on psychological well-being at T2. Specifically, the effect sizes for charitable donations between 1 and 100 USD (β = 0.14, *p* < 0.01) and greater than 100 USD (β = 0.23, *p* < 0.001) were both larger than the effect size of time volunteering between 1 and 10 h per month (β = 0.11, *p* < 0.05). Appau and Churchill (2019) investigated the relationships between volunteering/charitable donations, and subjective well-being, measured by life satisfaction. Volunteering and charitable donations were constructed as one variable indicating any volunteering or charitable donation. Using a national representative sample in the United Kingdom, this study found a positive association between individuals’ involvement in volunteering/charitable donations and life satisfaction.

Alongside the evidence from English literature, several studies provide a positive relationship between volunteering and Chinese individuals’ psychological well-being in Chinese literature (Zhang and Zhang, 2020; Zhang et al., 2021). For instance, Zhang and Zhang (2020) examined the effect of volunteer services on college students’ subjective well-being in China. Ninety-one college volunteers completed a survey prior to their volunteer activities as well as 1 year later upon the completion of the volunteer services, providing information about their subjective well-being in four dimensions: pleasant affect, unpleasant affect, life satisfaction, and domain satisfaction (Diener et al., 1999). The results of the *t*-test indicated that participants reported significantly higher levels of subjective well-being after the volunteer services, particularly in pleasant affect, life satisfaction, and domain satisfaction. In another study, Zhang et al. (2021) examined the relationship between volunteer work and subjective well-being using a representative sample of adults older than 45 years old in China. Participants’ subjective well-being was measured by subjective happiness (Diener, 2000). Among a total of 22,755 cases, 15.80% participated in volunteer work at least once in the past month. After accounting for a range of covariates, such as gender, age, health condition, marital status, the number of family members, and highest educational attainment, volunteer work was positively associated with individuals’ sense of happiness (Zhang et al., 2021). However, the current Chinese literature does not provide sufficient information regarding the relationship between charitable donations and psychological well-being.

In a word, both English and Chinese literature suggest a positive association between volunteering and psychological well-being. Though English literature provides some evidence about the positive association between charitable donations and psychological well-being, the amount of evidence is lacking in Chinese literature. Meanwhile, more studies focused on volunteering and the well-being of adults and older people (e.g., Morrow-Howell et al., 2003; McMunn et al., 2009; Choi and Kim, 2011), less on emerging adulthood (Zhang and Zhang, 2020). Emerging adulthood is the life stage that takes place when individuals are between 19 and 24 years old. The college years have been found to be a particularly important time for lifespan development, as this period is characterized by increasing independence and responsibility (Arnett, 2007; Costa et al., 2013; Marginson, 2017). Thus, the aim of this study is to examine the effects of volunteering and charitable donation on individuals’ psychological well-being on college students in China.

We hypothesize that college students’ psychological well-being is positively associated with their hours of volunteering and the amount of charitable donations. The hypothesis is guided by theories of altruism (Piliavin and Charng, 1990) and the warm-glow theory (Andreoni, 1990). Altruistic behaviors are individuals’ voluntary behaviors, which intentionally aim to benefit others (Bar-Tal, 1986). Altruism is an important factor of volunteering (Burns et al., 2006; Carpenter and Myers, 2010). Furthermore, the literature indicates that altruism has various beneficial effects on individuals (Lozada et al., 2011). Human being’s inherent need for social contact is highly related to altruism. Willingness and act of helping others are positively associated with the empathic ability (Domes et al., 2007), trust (Baumgartner et al., 2008), immune system (Pace et al., 2009), decreased stress level (Barraza and Zak, 2009), and prosocial behaviors (Brown et al., 2009). Therefore, although individuals do not intentionally expect any external rewarding by conducting altruistic behaviors (Bar-Tal, 1986), theories of altruism suggest that volunteer work rewards individuals in various forms, which could ultimately benefit their psychological well-being (Andreoni, 1988, 1990). Additionally, the warm-glow theory suggests that when individuals make charitable donations, they may gain benefits from the act of giving (Andreoni, 1990). Individuals gain internal satisfaction from giving, which promotes overall psychological well-being.

## Materials and Methods

### Data and Sample

Our data came from a web-based anonymous surveys that were administered to college students in China. To ensure a diverse sample, we selected universities that were geographically spread across China. A total of 12 universities from different regions of China (i.e., the north, south, east, west, and middle regions) were selected to participate in the study. In September 2020, we invited 2,229 junior and senior students from departments of social science of each college to participate the survey. Students received three notifications in total, including the initial invitation in late September and two reminders about the survey participation after 3 and 7 days of the initial invitation, respectively. Prior to start the survey, students were informed that the participation was voluntary and that they could choose to terminate their participation at any time during the survey. The survey was anonymous, not collecting any identifiable information from students. Upon completion of the survey, each participant could receive an incentive of 10 RMB (2 USD). The research protocol, including the informed consent process, was reviewed and approved by one of the co-authors’ institution. By early October 2020, 1,881 students completed and returned the survey. After omitting 10 incomplete surveys, our final analytic sample was 1,871 students, yielding a response rate of 84%.

### Measures

#### Dependent Variable

Students’ psychological well-being was measured using the 18-item version of the Psychological Well-being Scale (Ryff and Singer, 1996). This scale assessed six aspects of psychological well-being: autonomy, environmental mastery, personal growth, positive relations with others, purpose in life, and self-acceptance. Each aspect was measured by 3 items. Students indicated how strongly they agreed or disagreed with the statements using a 7-point Likert scale (1 = strongly agree; 7 = strongly disagree). Example items include: “The demands of everyday life often get me down;” “In general, I feel I am in charge of the situation in which I live;” and “I have confidence in my own opinions, even if they are different from the way most other people think.” The English version of the scale was translated into Chinese by two bilingual social work doctoral students and the translation was then verified by one bilingual social work faculty. We reverse-coded 10 opposite items and constructed students’ psychological well-being by adding up the responses to all items. The score of each subscale ranged from 3 to 21, and the scale of the whole scale ranged from 18 to 126. Higher scores indicated higher levels of psychological well-being. The Cronbach’s alpha of the scale was 0.88 in this study.

#### Independent Variables

Volunteering was measured according to students’ self-report of hours of volunteering in the past year. We first asked whether students had participated in any volunteer activity in the past year. If students provided an affirmative answer, follow-up questions were asked regarding the time they had spent on volunteer activities in hours as well as their frequency of volunteering (how many times). Students’ charitable donations were assessed following the same mechanism—students indicated whether they had made any charitable donations in last year as well as the frequency and total amount of their donations.

#### Covariates

We accounted for students’ demographic and socioeconomic characteristics in this study by collecting information on their age, gender (female vs. male), ethnicity (Han vs. other), residential status (rural, urban with prior rural, and urban), parents’ marital status (married, divorced, and others), parental level of education (elementary school or below, junior high school, high school, and college or above), number of family members, family income in the past year with log transformation, and social welfare status (yes vs. no). We also included the college-fixed effect to control for differences across universities.

### Analytic Plan

First, we undertook descriptive analysis to examine the distribution of main variables and the characteristics of the sample. Next, we conducted ordinary linear square (OLS) regression to estimate the net effects of volunteering and charitable donations on students’ psychological well-being, while accounting for demographic and socioeconomic characteristics. The analytic framework of this study posits that students’ levels of psychological well-being are determined by their hours in volunteering, the amount of charitable donations, as well as demographic and socioeconomic status. In addition, we conducted robust analyses by regressing psychological well-being subscales on the occurrence and frequencies of volunteering and charitable donations, while controlling for demographic and socioeconomic characteristics. All analyses were conducted using STATA software 16.0.

## Results

Table 1 displays the descriptive characteristics of the sample. The sample reported an average score of 81.75 on psychological well-being, with a range of 24-121 and a standard deviation (SD) of 12.30. The mean scores of the subscales are as followed: 12.95 (*SD* = 2.74) for autonomy, 13.83 (*SD* = 2.73) for environmental mastery, 14.22 (*SD* = 2.48) for personal growth, 13.87 (*SD* = 3.07) for positive relations with others, 13.47 (*SD* = 2.57) for purpose in life, and 13.41 (*SD* = 3.28) for self-acceptance. Students spent 24.73 h (*SD* = 32.23) on volunteer activities and 104.73 RMB (*SD* = 356.94) on charitable donations in the past year, on average. The sample had an average age of 20.62 years and about two-thirds (66.97%) of them were female. The residential status of the students was predominately urban (52.37%), followed by rural status (38.70%) and urban with prior rural status (8.93%). Most of the sample (89.36%) self-identified as Han ethnicity, and about 90% of students reported their parents were married. Close to 40% of students indicated parents’ highest education achievement was college or above, followed by junior high school (28.11%), high school (25.17%), and elementary school or below (6.90%). The average family income was 90,990 RMB (about 13,580 USD) in the past year. A quarter (25.28%) of students’ families had ever received social welfare, such as low-income assistance and food subsidies. The average number of family members of the sample was 3.87. Finally, the result of college composition suggests that each college occupied at least 2.46% of the whole sample with no college more than 11.54%.

**TABLE 1:** Descriptive statistics of main variables and covariates.

	Mean (*SD*)
Psychological wellbeing [24–121]	81.75 (12.30)
Autonomy [3–21]	12.95 (2.74)
Environmental mastery [3–21]	13.83 (2.73)
Personal growth [3–21]	14.22 (2.48)
Positive relations with others [3–21]	13.87 (3.07)
Purpose in life [3–21]	13.47 (2.57)
Self-acceptance [3–21]	13.41 (3.28)
Hours of volunteering [0–100]	24.73 (32.23)
Amount of charitable donations [0–6,000 RMB]	104.93 (356.95)
**Gender [%]**	
Female	66.97
Male	33.03
Age	20.62 (0.96)
**Residential status [%]**	
Rural	38.70
Urban with prior rural	8.93
Urban	52.37
**Grade [%]**	
Junior	60.72
Senior	39.28
**Ethnicity [%]**	
Han	89.36
Others	10.64
**Parent marital status [%]**	
Married	89.04
Divorced	6.89
Others	0.91
**Parent highest education achievement [%]**	
Elementary school and below	6.90
Junior high school	28.11
High school	25.17
College and above	39.82
Family income [RMB]	90,990 (1,22,030)
**Welfare status**	
No	74.72
Yes	25.28
Number of family members	3.87 (1.16)
Number of COVID-19 cases in province	14,264 (26,894)
**College composition[%]**	
College 1	7.11
College 2	9.57
College 3	6.25
College 4	10.85
College 5	10.15
College 6	7.06
College 7	6.41
College 8	11.54
College 9	11.12
College 10	2.46
College 11	6.89
College 12	10.58

*N = 1,871.*

We then conducted linear regression analyses to examine the net effects of volunteering and charitable donations on psychological well-being. Given the wide range of family income, hours of volunteering, and amount of charitable donations, these variables were transformed into natural log numbers prior to being entered into the regression analyses. Cases with values of 0 were assigned a 0.1 value before log transformation. Standardized coefficients are presented in Table 2. The results suggest that both hours of volunteering and the amount of charitable donations were positively associated with students’ psychological well-being, after controlling for all covariates. Specifically, every additional SD of log hours of volunteering was associated with a 0.10-SD increase in psychological well-being score (*p* < 0.001), and one SD of log amount of charitable donations was related to 0.08 SD higher psychological well-being score. The regression results support our hypothesis. Family income and the number of family members were related to psychological well-being as well. One SD increase in log family income led to a 0.05-SD increase in psychological well-being score (*p* < 0.05). On the contrary, one SD increased in the number of family members was associated with 0.06 SD lower psychological well-being score (*p* < 0.05). The adjusted R-square was 0.05. Additionally, according to results not shown but available upon request, the regression results by the occurrence and frequencies of volunteering and donations were similar to the ones reported here using hours and amount of volunteering and donations.

**TABLE 2:** Regression analysis of psychological well-being.

	β	S. E.	*P*
ln (hours of volunteering)	0.10	0.12	***
ln (amount of charitable donations)	0.08	0.08	***
Female	0.01	0.65	
Age	0.04	0.36	
**Residential status**			
Rural (reference group)	–	–	
Urban with prior rural	–0.01	1.05	
Urban	0.05	0.78	+
Junior	–0.01	0.71	
Han ethnicity	0.01	0.94	
**Parents’ marital status**			
Married	0.03	1.45	
Divorced	0.00	1.77	
Other (reference group)	–	–	
**Parents’ levels of education**			
Elementary school of below (reference group)	–	–	
Junior high school	–0.06	1.22	
High school	–0.02	1.28	
College or above	0.03	1.35	
ln (Family Income)	0.05	0.27	*
Welfare status	0.02	0.72	
Number of family members	–0.05	0.27	*
College fixed effects	Yes		
Adjusted R-square	0.05		

N = 1,871. +p < 0.10; *p < 0.05, ***p < 0.001.

We then conducted robust tests by regressing six psychological well-being subscales on independent and control variables following the same methods to produce the results presented in Table 2. For simplicity, we only present the results of the independent variables in Table 3. The findings are in line with those in Table 2. Both log hours of volunteering and log amount of charitable donations were significantly associated with the scores of psychological well-being subscales. Log hours of volunteering were positively associated with all but one aspects of psychological well-being: environmental mastery (β = 0.11, *p* < 0.001), positive relations (β = 0.10, *p* < 0.001), personal growth (β = 0.09, *p* < 0.001), self-acceptance (β = 0.09, *p* < 0.001), and purpose in life (β = 0.05, *p* < 0.05). Similarly, we observed positive associations between log amount of charitable donations and personal growth (β = 0.10, *p* < 0.001), positive relations (β = 0.06, *p* < 0.01), self-acceptance (β = 0.06, *p* < 0.05), autonomy (β = 0.05, *p* < 0.05), environmental mastery (β = 0.05, *p* < 0.05), and purpose in life (β = 0.05, *p* < 0.05).

**TABLE 3:** Regression analysis of psychological well-being subscales.

	Autonomy			Environmental mastery		
	β	*SE*	*P*	β	*SE*	*P*
ln (hours of volunteering)	0.00	0.03		0.11	0.03	***
ln (amount of charitable donations)	0.05	0.02	*	0.05	0.02	*

	**Personal growth**			**Positive relations**		

ln (hours of volunteering)	0.09	0.02	***	0.10	0.03	***
ln (amount of charitable donations)	0.10	0.02	***	0.06	0.02	**

	**Purpose in life**			**Self-acceptance**		

ln (hours of volunteering)	0.05	0.03	*	0.09	0.03	***
ln (amount of charitable donations)	0.05	0.02	*	0.06	0.02	*

N = 1,871. *p < 0.05, **p < 0.01, ***p < 0.001.

## Discussion

This study assessed the relationships between the hours of volunteering and the amount of charitable donations with college students’ psychological well-being in China. Consistent with our hypothesis, the findings suggest that both volunteering and charitable donations had significant associations with students’ psychological well-being. Specifically, students who had more hours of volunteer activities and higher amounts of charitable donations tended to report higher levels of psychological well-being than their counterparts. Further, the positive associations were consistently observed in six individual aspects of psychological well-being. It is noteworthy that the net effects of volunteering and charitable donations on psychological well-being subscales were slightly different. For instance, hours of volunteering had larger effects on environmental mastery and positive relations subscales than other aspects, while the amount of charitable donations had a larger effect on the personal growth subscale compared to others. Choi and Kim (2011) found that charitable donations had a more salient effect on older adults’ psychological well-being than time volunteering, while in our study, the positive association between volunteering with psychological well-being was slightly greater than the one between charitable donations and psychological well-being in college student sample. This discrepancy may be because that older adults, who retired after decades of career, are more financially capable to carry out charitable donations than college students who have not even started their career. Overall, the findings provide empirical evidence to theories of altruism and the warm-glow theory that volunteering and charitable donations could be self-rewarding, observed as increased levels of psychological well-being, for college students.

The findings of our investigation are consistent with the existing studies that found volunteering was positively related to the well-being of students in higher education (Schreier et al., 2013; Kim and Morgul, 2017; Ballard et al., 2018; Williamson et al., 2018). Our sample’s average hours of volunteering were approximately 25 h in the past year, and this number is close to the findings in Handy et al.’s (2010) study. Between 2006 and 2007, Handy et al. (2010) collected data from 9,482 college students in 12 countries, including China, regarding their volunteer activities. At least 600 Chinese college students participated in the study and reported 2.44 h of volunteering per month in the past year, which was equivalent to 29.28 h every year. Meanwhile, Handy et al.’s (2010) study also found that Chinese college students spent more hours volunteering than those in India and Japan.

Considering the positive association between volunteering and students’ well-being, academic institutions should encourage volunteer activities within students and consider integrating volunteering into the curriculum. In the past decades, Chinese universities and colleges have started to require a certain amount of volunteer activities as a part of the curriculum. Some have also provided for-credit service-learning courses. These requirements could strengthen students’ involvement in charitable activities and subsequently advance their overall well-being. Further, volunteering during school time, regardless of whether it is voluntary or required, may predict volunteering activities in adulthood (Hart et al., 2007; Tomkovick et al., 2008; Richard et al., 2016). Therefore, relevant curriculum and requirements may promote students’ well-being over a life course.

The designing of relevant curriculum and requirements should also consider the levels of intensity. While studies suggest volunteering is generally positive to psychological well-being, others also demonstrate that volunteering could be overwhelming sometimes and can decrease volunteers’ psychological well-being conversely (Shang et al., 2009; Choi and Kim, 2011; Feng and Zhao, 2011). By surveying a group of volunteer drivers of Beijing Summer Olympics in 2008, Shang et al. (2009) found that some volunteers experienced anxious and compulsive symptoms during their volunteer services. Additionally, Feng and Zhao (2011) pinpointed that the involvement of volunteer work during natural disasters or tragedies could produce volunteers’ secondary trauma and post-traumatic stress disorders (PTSD). In these cases, volunteer work could dampen volunteers’ mental health and psychological well-being. Thus, both universities and organizations should track students’ psychological and mental health status and provide appropriate counseling and mental health services as needed.

Several limitations to be aware of when interpreting the findings of this study. First, data were collected from students’ self-report, which leaves the data subject to intended and unintended reporting errors, such as social desirability bias. Social desirability bias denotes that study participants provide socially desirable responses rather than the responses that truly reflect their feelings (Grimm, 2010). Students might overreport their levels of psychological well-being to prevent stigma related to mental health issues (Corrigan et al., 2015). This could lead to over-estimated scores of psychological well-being as well as the magnitude of the relationships between volunteering and the amount of charitable donations with psychological well-being. Further research could reduce such reporting error via data triangulation, such as collecting information from peers, teachers, and family members. Second, using cross-sectional data, our study could only estimate associative relationships among variables of interests instead of causal ones. Future studies should utilize longitudinal research design to establish a temporal sequence among volunteering, charitable donations, and psychological well-being. Third, some unobserved variables that could influence psychological well-being were omitted in this study, such as personality traits (Kokko et al., 2013) and academic stressors (Verger et al., 2009). The absence of the omitted variables may affect the reported estimates. Finally, our sample was a group of college students who study social sciences in China. Even though the large sample size, as well as the diversity of universities, increase the generalizability of the findings to some extent, whether the findings could be representative of the college student population in China is unclear and needs further investigation.

## Conclusion

This study examines the relationships among volunteering, charitable donations, and psychological well-being using a sample of Chinese college students. The finding shows that both volunteering and charitable donations were positively associated with students’ psychological well-being. Volunteering displayed a larger association with students’ psychological well-being than charitable donations. The findings suggest that both volunteering and charitable donations lead to better psychological well-being as a self-rewarding of altruism behaviors. In spite of several limitations, this study provides a rationale for academic institutions to integrate social service activities into the curriculum as a potential tool to promote students’ psychological well-being.

## Data Availability Statement

The raw data supporting the conclusions of this article will be made available by the authors, without undue reservation.

## Ethics Statement

The studies involving human participants were reviewed and approved by the Institutional Review Board, Rutgers University-New Brunswick. Written informed consent for participation was not required for this study in accordance with the national legislation and the institutional requirements.

## Author Contributions

YG, YC, CH, and YT: conceptualization. YG, YC, CH, YT, CZ, and SZ: methodology and software, validation, formal analysis, investigation and data curation, and writing—original draft preparation. YG, CH, and YT: resources. All authors contributed to the article and approved the submitted version.

## Conflict of Interest

The authors declare that the research was conducted in the absence of any commercial or financial relationships that could be construed as a potential conflict of interest.

## Publisher’s Note

All claims expressed in this article are solely those of the authors and do not necessarily represent those of their affiliated organizations, or those of the publisher, the editors and the reviewers. Any product that may be evaluated in this article, or claim that may be made by its manufacturer, is not guaranteed or endorsed by the publisher.

## References

[B1] Andreoni J. (1988). Privately provided public goods in a large economy: the limits of altruism. *J. Public Econ.* 35 57–73. 10.1016/0047-2727(88)90061-8

[B2] Andreoni J. (1990). Impure altruism and donations to public goods: a theory of warm-glow giving. *Econ. J.* 100 464–477. 10.2307/2234133

[B3] Apinunmahakul A. Devlin R. A. (2008). Social networks and private philanthropy. *J. Public Econ.* 92 309–328. 10.1016/j.jpubeco.2007.07.005

[B4] Appau S. Churchill S. A. (2019). Charity, volunteering type and subjective wellbeing. *Voluntas* 30 1118–1132. 10.1007/s11266-018-0009-8

[B5] Arnett J. J. (2007). Emerging adulthood: what is it, and what is it good for? *Child Dev. Perspect.* 1 68–73. 10.1111/j.1750-8606.2007.00016.x

[B6] Awaworyi Churchill S. Mishra V. (2017). Trust, social networks and subjective wellbeing in China. *Soc. Indic. Res.* 132 313–339. 10.1007/s11205-015-1220-2

[B7] Ballard P. J. Hoyt L. T. Pachucki M. C. (2018). Impacts of adolescent and young adult civic engagement on health and socioeconomic status in adulthood. *Child Dev.* 90 1138–1154. 10.1111/cdev.12998 29359473

[B8] Barraza J. Zak P. (2009). Empathy toward strangers triggers oxytocin release and subsequent generosity. *Ann. N. York Acad. Sci.* 1167 182–189. 10.1111/j.1749-6632.2009.04504.x 19580564

[B9] Bar-Tal D. (1986). Altruistic motivation to help: definition, utility and operationalization. *Humboldt J. Soc. Relat.* 13 3–14.

[B10] Baumgartner T. Heinrichs M. Vonlanthen A. Fischbacher U. Fehr E. (2008). Oxytocin shapes the neural circuitry of trust and trust adaptation in humans. *Neuron* 58 639–650. 10.1016/j.neuron.2008.04.009 18498743

[B11] Bewick B. Koutsopoulou G. Miles J. Slaa E. Barkham M. (2010). Changes in undergraduate students’ psychological well-being as they progress through university. *Stud. High. Educ.* 35 633–645. 10.1080/03075070903216643

[B12] Black W. Living R. (2004). Volunteerism as an occupation and its relationship to health and wellbeing. *Br. J. Occup. Ther.* 67 526–532. 10.1177/030802260406701202

[B13] Boyle P. A. Barnes L. L. Buchman A. S. Bennett D. A. (2009). Purpose in life is associated with mortality among community-dwelling older persons. *Psychosom. Med.* 71 574–579. 10.1097/psy.0b013e3181a5a7c0 19414613 PMC2740716

[B14] Brim O. G. Ryff C. D. Kessler R. C. (2004). “The MIDUS National Survey: an overview,” in *How Healthy Are We? A National Study of Well-being at Midlife*, eds Brim O. G. Ryff C. D. Kessler R. C. (Chicago: University of Chicago Press), 1–34.

[B15] Brown S. L. Fredrickson B. L. Wirth M. M. Poulin M. J. Meier E. A. Heaphy E. D. (2009). Social closeness increases salivary progesterone in humans. *Horm. Behav.* 56 108–111. 10.1016/j.yhbeh.2009.03.022 19362559 PMC2699766

[B16] Burns D. J. Reid J. S. Toncar M. Fawcett J. Anderson C. (2006). Motivations to volunteer: the role of altruism. *Int. Rev. Public Nonprof. Market.* 3 79–91. 10.1007/BF02893621

[B17] Burris J. L. Brechting E. H. Salsman J. Carlson C. R. (2009). Factors associated with the psychological well-being and distress of university students. *J. Am. Coll. Health* 57 536–544. 10.3200/JACH.57.5.536-544 19254895

[B18] Carpenter J. Myers C. K. (2010). Why volunteer? Evidence on the role of altruism, image, and incentives. *J. Public Econ.* 94 911–920. 10.1016/j.jpubeco.2010.07.007

[B19] Choi N. G. Kim J. (2011). The effect of time volunteering and charitable donations in later life on psychological wellbeing. *Ageing Soc.* 31 590–610. 10.1017/S0144686X10001224

[B20] Corrigan P. W. Bink A. B. Fokuo J. K. Schmidt A. (2015). The public stigma of mental illness means a difference between you and me. *Psychiatry Res.* 226 186–191. 10.1016/j.psychres.2014.12.047 25660735

[B21] Costa H. Ripoll P. Sánchez M. Carvalho C. (2013). Emotional intelligence and self-efficacy: effects on psychological well-being in college students. *Spanish J. Psychol.* 16:E50. 10.1017/sjp.2013.39 23866247

[B22] Dickert S. Sagara N. Slovic P. (2011). Affective motivations to help others: a two-stage model of donation decisions. *J. Behav. Decis. Making* 24 361–376. 10.1002/bdm.697

[B23] Diener E. (2000). Subjective well-being: the science of happiness and a proposal for a national index. *Am. Psychol.* 55 34–43. 10.1037/0003-066X.55.1.34 11392863

[B24] Diener E. Suh E. M. Lucas R. E. Smith H. L. (1999). Subjective well-being: three decades of progress. *Psychol. Bull.* 125 276–302.

[B25] Domes G. Heinrichs M. Michel A. Berger C. Herpertz S. C. (2007). Oxytocin improves “mind-reading” in humans. *Biol. Psychiatry* 61 731–733. 10.1016/j.biopsych.2006.07.015 17137561

[B26] Feng S. S. Zhao J. B. (2011). Zhiyuanzhede xinlijiankangyuzhiyuanhuodongguanxi [Research of the relationship between volunteer activities and volunteer’s mental health]. *Chin. J. Soc. Med.* 28 119–124.

[B27] Froyum C. (2018). “They Are Just Like You and Me”: cultivating volunteer sympathy. *Symb. Interact.* 41 465–487. 10.1002/symb.357

[B28] Grimm P. (2010). “Social desirability bias,” in *Wiley International Encyclopedia of Marketing*, eds Sheth J. Malhotra N. (Hoboken: John Wiley & Sons Inc.). 10.1002/9781444316568.wiem02057

[B29] Handy F. Hustinx L. Kang C. Cnaan R. A. Brudney J. L. Haski-Leventhal D. (2010). A cross-cultural examination of student volunteering: is it all about resume building? *Nonprofit Volunt. Sect. Q.* 39 498–523. 10.1177/0899764009344353

[B30] Hart D. Donnelly T. M. Youniss J. Atkins R. (2007). High school community service as a predictor of adult voting and volunteering. *Am. Educ. Res. J.* 44 197–219. 10.3102/0002831206298173

[B31] House J. S. (1995). *Americans’ Changing Lives: Waves I and II, 1986 and 1989.* Ann Arbor: Inter-University Consortium for Political and Social Research.

[B32] Huppert F. A. (2009). Psychological well-being: evidence regarding its causes and consequences. *Appl. Psychol. Health Well Being* 1 137–164. 10.1111/j.1758-0854.2009.01008.x

[B33] Kaplan G. A. Shema S. J. Leite C. M. (2008). Socioeconomic determinants of psychological well-being: the role of income, income change, and income sources during the course of 29 years. *Ann. Epidemiol.* 18 531–537. 10.1016/j.annepidem.2008.03.006 18504142 PMC2771109

[B34] Keyes C. L. (2005). Mental illness and/or mental health? Investigating axioms of the complete state model of health. *J. Consult. Clin. Psychol.* 73 539–548. 10.1037/0022-006X.73.3.539 15982151

[B35] Kim J. Morgul K. (2017). Long-term consequences of youth volunteering: voluntary versus involuntary service. *Soc. Sci. Res.* 67 160–175. 10.1016/j.ssresearch.2017.05.002 28888284 PMC5612372

[B36] Kokko K. Tolvanen A. Pulkkinen L. (2013). Associations between personality traits and psychological well-being across time in middle adulthood. *J. Res. Pers.* 47 748–756. 10.1016/j.jrp.2013.07.002

[B37] Kubzansky L. D. Huffman J. C. Boehm J. K. Hernandez R. Kim E. S. Koga H. K. (2018). Positive psychological well-being and cardiovascular disease: JACC health promotion series. *J. Am. Coll. Cardiol.* 72 1382–1396. 30213332 10.1016/j.jacc.2018.07.042 PMC6289282

[B38] Lozada M. D’Adamo P. Fuentes M. A. (2011). Beneficial effects of human altruism. *J. Theor. Biol.* 289 12–16. 10.1016/j.jtbi.2011.08.016 21872608

[B39] Lyubomirsky S. King L. Diener E. (2013). The benefits of frequent positive affect: does happiness lead to success? *Psychol. Bull.* 131 803–855. 10.1037/0033-2909.131.6.803 16351326

[B40] Marginson S. (2017). Higher education, economic inequality and social mobility: implications for emerging East Asia. *Int. J. Educ. Dev.* 63 4–11. 10.1016/j.ijedudev.2017.03.002

[B41] McMunn A. Nazroo J. Wahrendorf M. Breeze E. Zaninotto P. (2009). Participation in socially-productive activities, reciprocity and wellbeing in later life: baseline results in England. *Ageing Soc.* 29 765–782. 10.1017/S0144686X08008350

[B42] Morrow-Howell N. Hinterlong J. Rozario P. A. Tang F. (2003). Effects of volunteering on the well-being of older adults. *J. Gerontol. Ser. B Psychol. Sci. Soc. Sci.* 58 S137–S145. 10.1093/geronb/58.3.S137 12730314

[B43] Musick M. A. Wilson J. (2003). Volunteering and depression: the role of psychological and social resources in different age groups. *Soc. Sci. Med.* 56 259–269. 10.1016/S0277-9536(02)00025-4 12473312

[B44] Pace T. W. Negi L. T. Adame D. D. Cole S. P. Sivilli T. I. Brown T. D. (2009). Effect of compassion meditation on neuroendocrine, innate immune and behavioral responses to psychosocial stress. *Psychoneuroendocrinology* 34 87–98. 10.1016/j.psyneuen.2008.08.011 18835662 PMC2695992

[B45] Piliavin J. A. Charng H. W. (1990). Altruism: a review of recent theory and research. *Ann. Rev. Sociol.* 16 27–65. 10.1146/annurev.so.16.080190.000331

[B46] Pressman S. D. Jenkins B. N. Moskowitz J. T. (2019). Positive affect and health: what do we know and where next should we go? *Ann. Rev. Psychol.* 70 627–650. 10.1146/annurev-psych-010418-102955 30260746

[B47] Richard D. Keen C. Hatcher J. A. Pease H. A. (2016). Pathways to adult civic engagement: benefits of reflection and dialogue across difference in higher education service-learning programs. *Michigan J. Commun. Serv. Learn.* 23 60–74.

[B48] Ryff C. D. Singer B. (1996). Psychological well-being: meaning, measurement, and implications for psychotherapy research. *Psychother. Psychosomat.* 65 14–23. 10.1159/000289026 8838692

[B49] Schreier H. M. Schonert-Reichl K. A. Chen E. (2013). Effect of volunteering on risk factors for cardiovascular disease in adolescents: a randomized controlled trial. *JAMA Pediatr.* 167 327–332. 10.1001/jamapediatrics.2013.1100 23440253

[B50] Shang X. H. Tang Y. X. Xu M. Pan X. (2009). Aoyunhuijiashiyuanxinlijiankangzhuangkuangyiganyuduice [Analysis of mental health of volunteer drivers of the Olympic Games and its countermeasures]. *Nurs. J. Chin. Peoples Liberation Army* 12 30–65.

[B51] Small D. A. Loewenstein G. Slovic P. (2007). Sympathy and callousness: the impact of deliberative thought on donations to identifiable and statistical victims. *Organ. Behav. Hum. Decis. Process.* 102 143–153. 10.1016/j.obhdp.2006.01.005

[B52] Tan T. Huang C.-C. Geng Y. Cheung S. P. Zhang S. (2021). Psychological well-being in Chinese college students during the COVID-19 pandemic: roles of resilience and environmental stress. *Front. Psychol.* 12:671553. 10.3389/fpsyg.2021.671553 34122262 PMC8192835

[B53] Tomkovick C. Lester S. Flunker L. Wells T. (2008). Linking collegiate service-learning to future volunteerism. *Nonprofit Manag. Leadersh.* 19 3–26. 10.1002/nml.202

[B54] Verger P. Combes J. B. Kovess-Masfety V. Choquet M. Guagliardo V. Rouillon F. (2009). Psychological distress in first year university students: socioeconomic and academic stressors, mastery and social support in young men and women. *Soc. Psychiatry Psychiatr. Epidemiol.* 44 643–650. 10.1007/s00127-008-0486-y 19096741

[B55] Wardle J. Cooke L. (2005). The impact of obesity on psychological well-being. *Best Pract. Res. Clin. Endocrinol. Metab.* 19 421–440. 10.1016/j.beem.2005.04.006 16150384

[B56] Williamson I. Wildbur D. Bell K. Tanner J. Matthews H. (2018). Benefits to university students through volunteering in a health context: a new model. *Br. J. Educ. Stud.* 66 383–402. 10.1080/00071005.2017.1339865

[B57] Wright T. A. Staw B. M. (1999). Affect and favorable work outcomes: two longitudinal tests of the happy-productive worker thesis. *J. Organ. Behav.* 20 1–23. 10.1002/(SICI)1099-1379(199901)20:1<1::AID-JOB885<3.0.CO;2-W

[B58] Wuthnow R. (1991). *Acts of Compassion.* Princeton: Princeton University Press.

[B59] Zhang W. C. Wu Y. Y. Yang H. L. (2021). Zhiyuangfuwu, nianlingchayiyuxingfugan [Voluntary service, age difference and subjective well-being]. *South Chin. J. Econ.* 40 106–124. 10.19592/j.cnki.scje.380058

[B60] Zhang Y. J. Zhang H. C. (2020). Zhiyuanfuwuduidaxueshen gzhuguanxingfuganyingxiangdeyanjiu [Examining the effect of volunteer services on college students’ subjective well-being]. *Weishengzhiyejiaoyu* 38 130–131.

